# Dr. Spittal on Pneumo-Thorax

**Published:** 1831-04-01

**Authors:** 


### EDINBURGH ROYAL INFIRMARY.

### PNEUMO-THORAX.

It appears that, in the year 1829, the Harveian Society of Edinburgh offered a prize for the best essay on "the Diagnostic Properties of the Stethoscope, illustrated by dissections." Mr. Robert Spittal, then physician's assistant, and now house-surgeon of the Royal Infirmary, was the successful candidate—and the result has been a volume or a treatise on auscultation, which now lies on our table. The author modestly enough acknowledges that "the present work presents little that is new to the experienced stethoscopist." He has, however, endeavoured to include all that has been made known on this subject down to the present time, and as the body of cases and dissections may fairly be presumed to have occurred in the Royal Infirmary (though this statement is not officially made) the work forms a valuable manual for the young auscultator. We have prefaced the following case of pneumo-thorax by these few remarks, as we may not have an opportunity of reviewing Mr. Spittal's book in the regular way—the work being an elementary compilation, and therefore insusceptible of analysis.

*Case.* "G. I., a man, æt. 38, July 27, 1830.—Has a troublesome cough, which is usually worse during the night, but he has no pain in the chest, except at times when the cough is very severe. Expectoration scanty and muco-purulent. He can lie on either side, but says he is easiest when lying on the right. Complains of general weakness, and perspires profusely during the night. Bowels in general rather slow. Tongue white. Appetite impaired. Great thirst. Pulse 120. Skin hot. Emaciation considerable.

Reports that his complaints commenced about six weeks ago, after exposure to cold and wet.

28th. Sound of vesicular respiration heard, generally faint, over both sides of the chest. Sound on percussion good.

August 7th. Yesterday he suddenly became affected with severe pain in the left shoulder, and extending down that side of chest, accompanied with difficulty of breathing.

21st. Percussion over the anterior part of left side of chest, when lying in bed, louder than over the right side; and metallic resonance is heard there.

23d. Metallic resonance heard, after coughing, over nearly the whole of the left side, anteriorly and laterally, from 1st to 11th rib. Amphorique respiration is likewise heard in the same situation. Succussion of the chest produces a distinct sound of fluctuation. The left side of the chest is much protruded, and the intercostal spaces are widened. Percussion over this side of chest produces a tympanitic sound, even to a low point, when he is sitting up.

Slight tapping upon the chest produces metallic resonance, which is distinctly audible on the application of the stethoscope.

Respiration frequent, but he experiences very little uneasiness. Pulse frequent and soft.

24th. Metallic tinkling heard occasionally on left side of chest, without any effort on his part. Metallic resonance heard as before, after coughing, and, for the most part, after every word or syllable he utters.

Action of the heart cannot be felt by the hand, but is distinctly heard between cartilages of the 3d and 4th ribs on right side.

Sound of respiration heard over right side rather puerile. Cough not very troublesome; expectoration muco-purulent. Pulse 112, small and soft. Skin hot. Lies occasionally on the left side, but prefers lying on the right, which he most usually does. Emaciation increasing.

30th. Febrile symptoms abated. Pulse 92, soft. Respirations 24 in a minute. Cough less. Still prefers lying on the right side. Has occasional paroxysms of dyspnœa. Metallic resonance and amphorique respiration as before. States that a few days ago he heard a "ringing sound" in his chest, after moving himself.

September 5th. Has been daily becoming more emaciated and feeble. Metallic resonance heard to-day as before.

6th. Died about 2 p. m.

8th. *Examination.* Left side of chest still much protruded, and tympanitic on percussion. Metallic resonance is easily produced, by tapping smartly with the fingers on the sternum; and it is distinctly heard by the application of the stethoscope to the left side of the chest at the same time. Succussion elicits the sound of fluctuation; and the sound produced by percussion is altered by change of position.

On puncturing the anterior part of the left side of the chest, air was perceived to issue; and, on opening the chest, a large space was found occupied by air, corresponding to the part which had elicited the clear sound on percussion.

There were likewise about four pounds of sero-purulent fluid, containing many portions of coagulated lymph in the left side of the chest. The pleura costalis was coated over with a regular soft, slightly adherent, layer of lymph.

The left lung was compressed to a small bulk, and lay close to the mediastinum and spine, but, except at the upper and back part, it did not adhere very firmly. The pleura pulmonalis was very slightly coated with lymph.

At the apex of the lung there was an irregular opening, capable of admitting the point of the finger. This orifice was found to communicate with a large irregular tubercular cavity, which communicated with the bronchial tubes.

The lung contained tubercular matter, in different stages, and was generally in the usual state of carnification which compressed pulmonary tissue is found to assume.

The heart and its appendages lay much to the right of their natural situation. The valves of the left side of the heart were slightly diseased.

The right lung was somewhat emphysematous, and contained a few groupes of tubercles in different stages, but there were no excavations in this lung.

*Remarks.*—It is probable that in this case the pneumo-thorax took place on August 7th, but it was not proved until percussion and auscultation had been made use of on the 21st. The metallic resonance heard after coughing and speaking proved the presence of air in the chest. The metallic tinkling, occurring without any effort, was probably caused by the bursting of a considerable air-bell, so that this, as well as the succussion, proved the existence of air and liquid in the chest.

The amphorique respiration indicated the existence of a communication with the bronchial tubes.

The respiration was rather puerile in the right lung, and it was found only to contain a few tubercles.

The site of the heart accorded with the place where its action had been heard during life.

In this case the metallic resonance was produced equally by percussion on the chest during life as after death; proving distinctly, that this phenomenon is dependent upon the resonance which takes place in any considerable cavity containing air."*

---

* Spittal on Auscultation, p. 235-9.

